# Topical application of Acheflan on rat skin injury accelerates wound healing: a histopathological, immunohistochemical and biochemical study

**DOI:** 10.1186/s12906-015-0745-x

**Published:** 2015-06-30

**Authors:** Jamila Alessandra Perini, Thais Angeli-Gamba, Jessica Alessandra-Perini, Luiz Claudio Ferreira, Luiz Eurico Nasciutti, Daniel Escorsim Machado

**Affiliations:** Laboratório de Pesquisa de Ciências Farmacêuticas, Unidade de Farmácia, Centro Universitário Estadual da Zona Oeste, Av. Manoel Caldeira de Alvarenga, 1203, Campo Grande, 23070-200 Rio de Janeiro, RJ Brazil; Programa de Pós-Graduação em Saúde Pública e Meio Ambiente, Escola Nacional de Saúde Pública, Fundação Oswaldo Cruz, Rio de Janeiro, RJ Brazil; Instituto de Ciências Biomédicas, Universidade Federal do Rio de Janeiro, Rio de Janeiro, RJ Brazil

**Keywords:** Acheflan®, *Cordia verbenacea*, Wound healing, Skin, Collagen, VEGF

## Abstract

**Background:**

Dermal wound healing involves a cascade of complex events including angiogenesis and extracellular matrix remodeling. Several groups have focused in the study of the skin wound healing activity of natural products. The phytomedicine Acheflan®, and its main active constituent is the oil from *Cordia verbenacea* which has known anti-inflammatory, analgesic and antimicrobial activities. To our knowledge, no investigation has evaluated the effect of Acheflan® in an experimental model of skin wound healing.

The present study has explored the wound healing property of Acheflan® and has compared it with topical effectiveness of collagenase and fibrinolysin by using Wistar rat cutaneous excision wound model.

**Methods:**

Animals were divided into four groups: untreated animals are negative control (NC), wounds were treated topically every day with Collagenase ointment (TC), with Fibrinolysin ointment (TF) and with cream Acheflan (TAc). Skin samples were collected on zero, 8th and 15th days after wounding. The healing was assessed by hematoxylin-eosin (HE), picrosirius red, hydoxyproline content and immunohistochemical analysis of the vascular endothelial growth factor (VEGF) and matrix metalloprotease-9 (MMP-9). Statistical analysis was done by ANOVA and Student *t*-test (*p* < 0.05).

**Results:**

The histological analysis HE of wound in the TAc group was more efficient because it was possible to observe the complete remodeling of the epidermis indicating the regression of lesions compared with the NC. The evaluation of picrosirius staining has demonstrated a significant increase of collagen distribution in the TC and TAc treatments compared with NC and TF groups. These results are corroborated with hydroxyproline content. Skin TC and TAc treated rats have showed an increase of VEGF and MMP-9 compared with NC and TF groups. All parameters were significant (*P* < 0.05).

**Conclusion:**

The phytomedicine Acheflan® (oil of *Cordia verbenacea*) and TC possess higher therapeutic properties for wound healing compared with TF. These ointments seem to accelerate wound healing, probably due to their involvement with the increase of angiogenesis and dermal remodeling.

## Background

Dermal wound healing is a physiological process involving several overlapping stages that could include inflammation, formation of granulation tissue, reepithelialization, extracellular matrix (ECM) formation and remodeling. Loss of skin integrity through trauma, injury and chronic ulcerations may result in homeostasis imbalance and in significant failure [1]. Wounds are major concerns for the medical staff and seriously reduce the quality of life of the patient. Furthermore, skin wound is a public health problem with high cost and ineffective treatment [2]. Thus, the attempt to quickly close the skin lesions with ideal functional and aesthetic results would be the goal of clinical treatment [3].

Nowadays, there has been extensive scientific interest in pharmacological evaluation on the biological properties of phytotherapeutics products [4, 5]. In addition, several studies have focused in evaluating the wound healing activity of medicinal plants, such as *Cleome viscose* [6], *Crotalus adamanteus* [7], *Blumea balsamifera* [8], *Salvia miltiorrhiza* [9], *Bacopa monniera* [10], *Vitis Vinifera* [11] and *Morinda citrifolia* [12]. Using an excision wound model in rat, Nayak and colleagues described that extract of *Punica granatum* promotes faster wound healing from hydroxyproline analysis and histological studies [13]. In this way, experimental models have been developed and have significant improved our knowledge of wound repair because they can be easily accessed to test the efficacy of different treatments [14, 15].

*Cordia verbenacea* is a Brazilian plant used to producing a phytomedicine, which is the main active constituent of the product Acheflan® developed in Brazil and approved by ANVISA (Agência Nacional de Vigilância Sanitária) in 2004 for the management of trauma, tendinopathy and myofacial pain [4]. In Brazil, the companies need to prove the safety efficacy, quality and safety based on scientific information of phytomedicines because they are registered as drugs [16]. Previous phytochemical study performed with *C. verbenacea* had demonstrated the main constituents of the essential oil identified by gas chromatography/mass spectrometry (GC-MS) (30 % of α-pinene, 25 % of *trans*-caryophyllene, 10 % of aloaromadendrene and 5 % of α-humulene) [17]. In addition, Chaves and colleagues [18] quantified by GC-MS the main active constituent isolated from Acheflan®, the α-humulene, and 30 min after topical administration of Acheflan®, the amount of α-humulene absorbed in the ear of the mice was about 2 μg/ear. Furthermore, it has been previously reported that the *C. verbenacea* has anti-inflammatory, analgesic and antimicrobial activity [18–24] and low toxicity [19].

To our knowledge, no investigation has evaluated the effect of Acheflan® in skin injury and our hypothesis was that its use may accelerate the stages of wound healing process. Thus, the purpose of this *in vivo* study was to evaluate topical effectiveness of Acheflan® on tissue formation, reepithelialization, angiogenesis and, collagen deposition under cutaneous injury and compared with traditional agents (collagenase and fibrinolysin).

## Methods

### Experimental animal model

Sprague–Dawley rats were used in the accomplishment of the full-thickness excisional wound model using the method described by [25]. 8 weeks old animals and each weighing 250–300 g were housed individually in individual polyethylene cages, and were kept at a constant temperature (25 °C) under a 12-h light/dark cycle with free access to food and water in the Bioterium of Universidade Estadual da Zona Oeste – UEZO (Rio de Janeiro, Brazil). The experimental procedure was approved by the UEZO Institutional Animal Care and Use Committee (CEUA), protocol code CEUA-UEZO-002/2013, and all experiments were conducted in performed with the ethical guidelines from the CEUA.

After the induction of general anesthesia with intraperitoneal ketamine (90 mg/kg) and xylazine (10 mg/kg), the rats’ dorsal regions were shaved and cleaned with ethanol 70 %. The circular full-thickness excision wound was made with a biopsy punch of 20 mm in diameter. The wound was left undressed to the open environment.

### Phytomedicine

Acheflan® is a phytomedicine developed in Brazil approved by the local authority ANVISA, Brazilian FDA-like agency, at 2004. This product is a topical drug for the management of trauma, tendinopathy and myofacial pain developed from the Brazilian medicinal plant *Cordia verbenacea DC (Boraginaceae)*. Acheflan® cream (TAc) containing 0.5 % of *C. verbenacea* essential oil and 2.5 % of α-humulene provided by Aché Laboratory, Brazil.

### Wound model and topical treatment

Immediately after surgical excision the rats were randomly divided into four groups of each six animals: untreated animals are negative control (NC), wounds daily treated topically with Collagenase ointment (TC), wounds daily treated topically with Fibrinolysin (1 U fibrinolysin, 666 U DNAse and 10 mg chloramphenicol) ointment (TF) and wounds daily treated topically with phytomedicine cream TAc. The rats were euthanized with overdose of anesthesia at 8 and 15 days after wounding. The skin wound samples were collected in each time point: zero (n = 6), 8 days (n = 3) and 15 days (n = 3). The granulation tissue formed on the injury was excised leaving a 5 mm margin of normal skin for histopathological assessment and determination of hydroyproline.

### Histopathologic analysis

The full thickness wound tissues, including the adjacent skin, were fixed immediately in formalin, paraffin-embedded and cut into 4-μm-thick sections. Part of the sections were stained with Harris hematoxylin and eosin (HE), and examined microscopically by two blinded observers using a 40× objective lens of a light microscope (Nikon, Tokyo, Japan) connected to a digital camera (Coolpix 990; Nikon). To estimate the degrees of wound healing a histological score was used to determine the dermal and epidermal regeneration and granulation tissue formation, as described by Kim and colleagues [26]. Additional sections were stained with Picrosirius red for observation of collagen fibers distribution through the calculus of the percentage of the marked area in reddish-yellow by field by using the Image Pro Plus 4.5.1 (Media Cybernetics, Silver Spring, MD).

### Immunohistochemistry and morphometric evaluation

The other paraffin-embedded tissue sections were placed on silane-treated slides, and maintained at room temperature. After dewaxing, the sections were treated with a solution of 3 % H2O2 in 0.01 mol/L phosphatebuffer saline (PBS), pH 7.5, to inhibit endogenous peroxidase activity. The slides were then immersed in 10 nmol/L citrate buffer (pH 6.0) and heated in a microwave oven for 5 min to retrieve masked antigens, to reduce nonspecific antibody binding; the sections were then incubated with PBS containing a 10 % solution of normal goat serum and 5 % bovine serum albumin for 30 min. Sections were incubated with the following antibodies: monoclonal antibody against VEGF SC-7269 (Santa Cruz Biotechnology, Santa Cruz, CA) at 1:100 dilution and polyclonal antibody against MMP-9 SC-6840 (Santa Cruz Biotechnology, Santa Cruz, CA) at 1:200 dilution. Incubations were carried out overnight and then revealed using LSAB2 Kit HRP, rat (Dako-Cytomation, Carpinteria, CA) with diaminobenzidine (3,3’-diaminobenzidine tablets; Sigma, St. Louis, MO) as the chromogen and counterstained with hematoxylin. For each case, negative control slides consisted of sections incubated with antibody vehicle or no immune rabbit or mouse serum. Ten fields of an immunostained section (VEGF and MMP-9) were chosen at random and captured from each specimen. Quantification was assessed on captured highquality images (2048 × 1536 pixels buffer) using the Image Pro Plus 4.5.1 (Media Cybernetics, Silver Spring, MD). Data were stored in Adobe Photoshop, version 3.0, to enable uneven illumination and background color to be corrected. Histologic scores (H) for VEGF and MMP-9 were calculated using the formula H = ΣPi, where i is the intensity ranging from 0 (negative cells) to 3 (deeply staining cells) and P is the percentage of staining cells for each given i, with *P* values of 1, 2, 3, 4, and 5 indicating <15 %, 15–50 %, 50–85 %, >85 %, and 100 % positive-staining cells, respectively. The staining result was expressed as mean ± standard deviations.

### Biochemical analyses of the newly formed skins

The hydroxyproline, the basic constituent of collagen, was taken as a marker of collagen synthesis. The granulation tissue from control and treated groups was dried at 60–70 °C for 24 h and weighed to determine the dry granulation tissue weight. Pieces of dried tissue were hydrolysed in 6 N HCl at 120 °C for 18 h in sealed tubes. The hydrolyzed samples were adjusted to pH 7.0 and were subjected to chloramines-T oxidation for 20 min. The reaction was terminated by addition of 3.15 M perchloric acid and para-dimethylaminobenzaldehyde at 60 °C to develop a pink color [27]. Absorbance was measured at 557 nm using a spectrophotometer. The procedure was done in triplicate for all samples, in each time point (zero, 8 and 15 days) and the hydroxyproline content was determined against a standard curve of hydroxyproline.

### Statistical analysis

One-way analysis of variance (ANOVA) was carried out to identify the differences between treated groups and controls. Statistical comparisons between variables were performed with Student *t*-test. The level of significance for significant difference between groups was set at *P <*0.05 in all analyses.

## Results

**Fig. 1:**
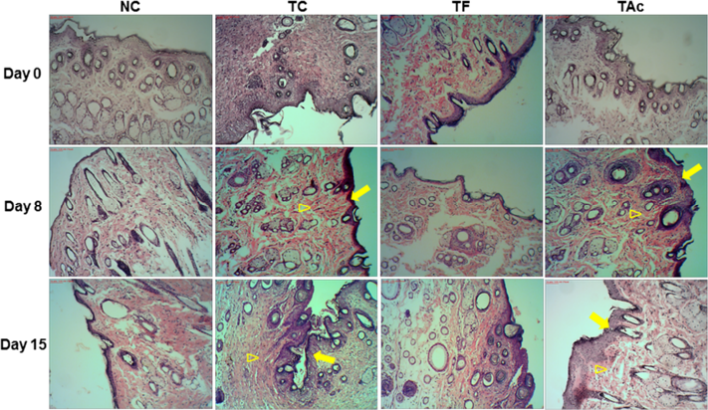
The histological evaluation of the skin flaps revealed by HE coloration (10x). Microscopic examination of TC and TAc groups indicated regression of the lesions with better epithelialization (arrows) and more effective re-organization of the dermis (arrowhead) compared to the NC and TF on 8^th^ and 15^th^ days after injury

Histologic wound scoring was conducted in a blinded fashion using the dermal and epidermal regeneration and the granulation tissue thickness, as described by Kim and colleagues [26]. In both observations, the histological analysis of wound in the treated groups at days 8 and 15 showed that wounds displayed better epithelialization and more effective re-organization of the dermis when compared with the control group (Fig. 1). When compared between the treated groups, the TAc was more efficient because it was possible to observe that the complete remodeling of epidermis indicated the regression of the lesions.

**Fig. 2:**
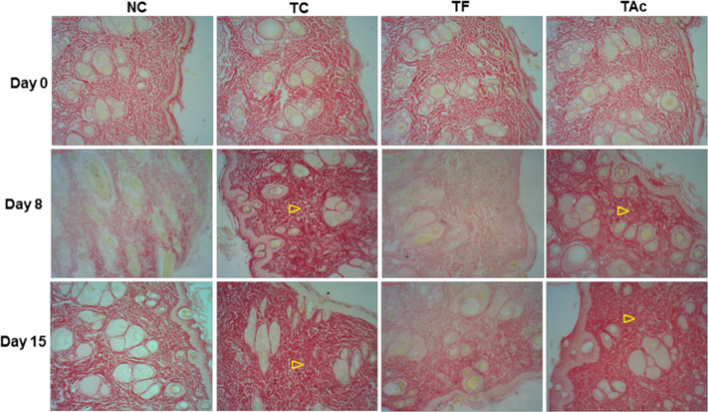
The evaluation of Picrosirius staining was made to recognize the total density of collagen. In each cut we analyzed the percentage of area occupied by collagen fibers (reddish-yellow). The distribution of collagen was more intense mainly in TC and TAc groups (arrowhead) on 8^th^ and 15^th^ days after injury

The evaluation of picrosirius staining demonstrated a significant increase of collagen distribution in the TC and TAc treatments compared with NC and TF groups. Already on day 8, it was possible to observe the intense reddish-yellow coloration of the collagen on TC and TAc, indicates that especially these treatments have contributed to the greater synthesis of these fibers (Fig. 2). These observations were confirmed by the percentage of area occupied by collagen fibers in each cut (Table 1).

**Table 1:** Histologic scores of collagen fibers, VEGF and MMP-9 in studied groups

Score (%)	Group	Day 0	Day 8	Day 15
Collagen fibers	NC	60.3 ± 0.9	16.5 ± 1.4	59.9 ± 1.6
	TC	60.3 ± 0.9	70.6 ± 1.2^b, c^	71.3 ± 1.6^b, c^
	TF	60.3 ± 0.9	10.9 ± 1.6^b^	20.2 ± 1.4^b^
	TAc	60.3 ± 0.9	68.9 ± 1.2^b, c, d^	67.7 ± 0.8^b, c, d^
*P* value^a^		1.0	0.0001	0.0001
VEGF	NC	1.5 ± 0.7	19.5 ± 2.1	2.6 ± 1.0
	TC	1.5 ± 0.7	28.2 ± 1.5^b, c^	15.5 ± 1.3^b, c^
	TF	1.5 ± 0.7	18.6 ± 0.8	12.0 ± 1.2^b^
	TAc	1.5 ± 0.7	26.4 ± 1.8^b, c, d^	17.3 ± 1.1^b, c, d^
*P* value^a^		1.0	0.0001	0.0001
MMP-9	NC	0.9 ± 0.8	10.2 ± 0.9	8.3 ± 1.1
	TC	0.9 ± 0.8	47.8 ± 1.9^b, c^	10.8 ± 1.9^b^
	TF	0.9 ± 0.8	11.9 ± 1.4^b^	10.9 ± 1.7^b^
	TAc	0.9 ± 0.8	17.1 ± 0.8^b, c, d^	11.4 ± 0.6^b^
*P* value^a^		1.0	0.0001	0.001

*NC* is negative control, *TC* is treated topically with Collagenase ointment, *TF* is treated topically with Fibrinolysin and *TAc* is treated topically with phytomedicine cream Acheflan. Day zero, 8^th^ and 15^th^ days after injury. Values are mean ± standard deviations. ^a^Anova test. ^b^Significant difference when compared to NC group (Student *t* test, *P* < 0.05). ^c^Significant difference when compared to TF group (Student *t* test, *P* < 0.05). ^d^Significant difference when compared to TC group (Student *t* test, *P* < 0.05)

Similar results were shown on biochemical analysis of hydroxyproline levels, the basic constituent of collagen. In the TC and TAc, the hydroxyproline content of dry granulation tissue was significantly higher compared with NC and TF on day 8 (Table 2). These results suggest that TC and TAc have strong wound healing potential.

**Table 2:** Hydroxyproline levels in wound areas of the all treatment groups

Group	Day 0	Day 8	Day 15
NC	66 ± 8.3	67 ± 5.3	115 ± 2.7
TC	63 ± 1.9	86 ± 0.8^b, c^	122 ± 2.2
TF	59 ± 1.6	65 ± 1.3	120 ± 5.7
TAc	64 ± 2.7	107 ± 9.1^b, c, d^	127 ± 10.5
*P* value^a^	0.087	0.0001	0.206

*NC* is negative control, *TC* is treated topically with Collagenase ointment, *TF* is treated topically with Fibrinolysin and *TAc* is treated topically with phytomedicine cream Acheflan. Day zero (n = 6), 8^th^ (n = 3) and 15^th^ (n = 3) days after injury. Values are mean ± standard deviation from six animals in each group at day zero and three animals in each group at 8^th^ and 15^th^ days after injury. ^a^Anova test. ^b^Significant difference when compared to NC group (Student *t* test, *P* < 0.05). ^c^Significant difference when compared to TF group (Student *t* test, *P* < 0.05). ^d^Significant difference when compared to TC group (Student *t* test, *P* < 0.05)

**Fig. 3:**
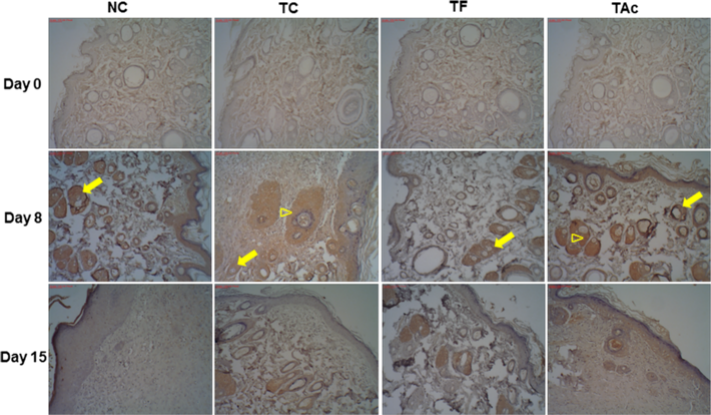
Photomicrograph from immunostained with an antibody against VEGF in control and treated groups. The dermis in both groups exhibits positive VEGF immunostaining (arrows); in TC and TAc the immunoreaction is higher, especially in day 8 (arrowheads)

The distribution of VEGF, which is one of the most prominent angiogenic markers, was detected focally on the dermis in both control and treated groups (Fig. 3). On day 8, the immunoreactivity was higher again in TC and TAc and it was almost similar in the other groups. On the day 15, the distribution of VEGF was reduced in all treated groups, but in TC and TAc there are less reduction, while a low expression was seen in the NC. The histologic scores of VEGF were statistically higher in TC and TAc, as shown by the morphometry evaluation (Table 1).

**Fig. 4:**
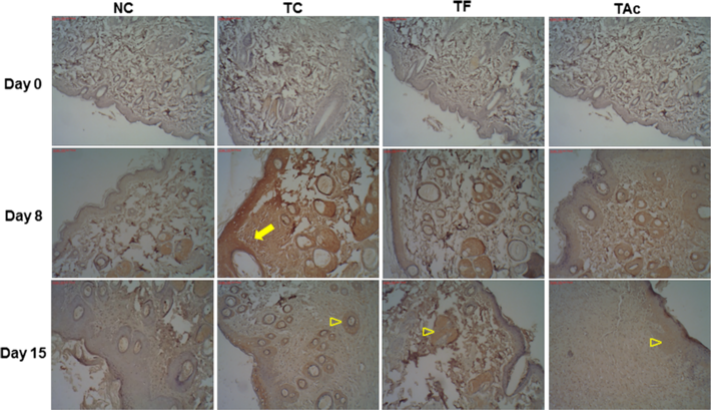
Immunohistochemical staining with MMP-9 in control and treated groups. The pattern of distribution of the MMP-9 staining is similar as with the VEGF study, but anti–MMP-9 antibody immunoreactivity was more intense in TC on day 8 (arrows); on the day 15, the immunoreactions were reduced and no differences were observed among the treated groups (arrowheads)

The MMP-9 immunodistribution was similar to that of VEGF, but was more intense in TC on day 8 (Fig. 4). At this time, the reactivity of the MMP-9 was very increased in TC, which strong labeling in all the tissue. As observed in the VEGF study, the distribution of MMP-9 was reduced on the day 15, and no differences were observed among the treated groups. Comparing the different groups, MMP-9 histologic scores were higher, particularly in TC on day 8 (Table 1).

## Discussion

In this study we propose for the first time that Acheflan® possess higher therapeutic properties for wound healing compared with TF and the effect was similar to TC. We have demonstrated a better tissue formation and reepithelialization, an increased distribution of collagen deposition and significantly enhanced angiogenic marker in the cutaneous injuries treated with TC and Acheflan®.

Skin wound healing is a complex physiological process that involves multiple tissue and cell types, and usually the wound progresses toward homeostasis through steps involving inflammation, new tissue formation, and tissue remodeling [28]. However, cutaneous wound healing is a major interest for the public health sector because the skin wounds affect a large number of patients, seriously reducing their quality of life, requiring extended hospitalization time, and accounts for a significant amount of healthcare expenditures. Furthermore, the scientific information about the potential effect of topical agents on skin wound healing is limited [29]. Natural products have consistently been an important source of therapeutic agents, therefore, we have evaluated and compared topical effectiveness of collagenase and fibrinolysin with phytomedicine Acheflan® on cutaneous injury.

Proteolytic enzymes have been used for wound debridement for many years and the two most widely used enzymes in the world are fibrinolysin/DNAse and collagenase [30–33]. The collagenases, an enzymatic debriding agent, act by degrading native helical collagen fibrils [34]. Recently, Tallis and colleagues [33] showed that collagenase ointment is tolerable and clinically effective in achieving the removal of nonviable tissue in the preparation of a healthy wound bed. In addition, a study on infected accidental or surgical wounds, debridement using fibrinolysin/ DNAse was reportedly effective [35].

The main active constituent from the phytomedicine Acheflan® is the oil from *C. verbenacea*, and its topical anti-inflammatory and antinociceptive properties have already been reported [18, 20, 23, 24]. Acheflan® was approved by ANVISA in Brazil for the management of tendinopathy, myofacial pain and trauma [4]. As far as we know, the present work is the first study to focus on the effect of Acheflan® in wound-healing model, and we have showed that this phytomedicine had a role in the early wound healing processes. This effect displayed may be attributed to the compounds isolated from *C. verbenacea*, sesquiterpene α-humulene, which has revealed important anti-inflammatory and antinociceptive properties [18, 20, 23, 24]. In addition, previous data with Acheflan® cream showed that the α-humulene is completely and fast absorbed when applied topically [18].

Collagen has a well-established function in early wound healing, and is the main component which strengthens and supports extra cellular tissue, it is composed of amino acid, hydroxyproline, which has been used as a biochemical marker for tissue collagen [36, 37]. During the proliferative phase, type III collagen is secreted by migrating and proliferating fibroblasts, in and around the wound, and this collagen is essential for creating the provisional matrix during this phase. Type I collagen is also vital during the maturation period and the most prevalent collagen in uninjured skin [38]. Acheflan® has demonstrated a significant increase in the hydroxyproline content and collagen distribution of the granulation tissue after 8 days of injury indicating increased collagen turnover. Similar results were reported with increase hydroxyproline content around 2 times using excision wound model in rats treated with extract of *Punica granatum* [13], *Vitis vinifera* and *Vaccinium macrocarpon* [39], *Allamanda cathartica* [40], *Ixora coccinea* [41] and *Cleome viscose* [6].

We used the immunohistochemical technique to observe the angiogenesis process through VEGF marking in our experimental model. The VEGF is an angiogenic peptide produced by endothelial cells, macrophages, and many other cells, being an excellent marker for endothelial cells [42]. In the present study, the TC and Acheflan® exhibited the most intense distribution of VEGF in day 8th, indicating that this factor is mainly stimulated in the early period of wound healing. This is in agreement with the findings that many processes are involved in the early period of wound healing and require action of many factors to facilitate cell movement, granulation tissue formation and angiogenesis [43–45].

On the other hand, changes in the ECM associated with disease states may arrest progression of the sequence of stages, critical for wound healing. Exorbitant production of a collagenous matrix can be also questionable, resulting in cutaneous scarring that produce esthetic problem, functional damage, and discomfort [46]. In our study there is an interesting difference between MMP-9 distribution in TC and Acheflan® on day 8, with distribution in TC group extensively higher. We know that MMP-9 have important function in the angiogenesis process in dermal remodeling, but exacerbated levels seem to be negatively implicated in the tissue degradation. In the same way, Kurtz and Oh [47] also demonstrated that the aberrant ECM expression may provide to pathologic wound responses.

We should consider that human skin differs from rat skin, and the important difference is that human skin heals preferentially by re-epithelialisation, while rat skin heals mainly by wound contraction [14]. We acknowledge the limitations for translational relevance of our experimental study; however, skin lesions in animal models are pertinent because provide significant contributions to advances in the treatment of skin wounds. Despite the limitations of the experimental model, we have showed higher topical effectiveness of Acheflan® on tissue formation, reepithelialization, angiogenesis, collagen deposition under cutaneous injury compared with TF.

## Conclusion

In conclusion, our findings have demonstrated that Acheflan® accelerates wound healing in skin rat model, probably due to its involvement with the increase angiogenesis and dermal remodeling.

## Abbreviations

CEUA: Institutional Animal Care Committee
DNAse: Desoxirribbonuclease
ECM: Extracellular matrix
GC-MS: Gas chromatography/mass spectrometry
H: Histologic scores
HE: Hematoxylin-eosin
MMP-9: Matrix metalloprotease-9
NC: Negative control
PBS: Phosphatebuffer saline
TC: Collagenase ointment
TF: Fibrinolysin ointment
TAc: Cream Acheflan
UEZO: University State of West Zone
VEGF: Vascular endothelial growth factor
